# Social Support Mediates the Association between Attachment Style and Psychological Distress during COVID-19 in Israel

**DOI:** 10.3390/v14040693

**Published:** 2022-03-27

**Authors:** Tal Adar, May Davidof, Odelia Elkana

**Affiliations:** School of Behavioral Science, Tel Aviv- Yaffo Academic College, Tel Aviv-Yafo 6818211, Israel; taladar134@gmail.com (T.A.); maydavidof2@gmail.com (M.D.)

**Keywords:** COVID-19, psychological distress, depression, anxiety, attachment style, social support

## Abstract

Aim: The aim of this study was to examine the relationships between attachment style, social support, and psychological distress (i.e., depression and anxiety) during the COVID-19 lockdown of the third wave in Israel. Specifically, we examined whether social support mediates the well-documented relationship between attachment style and psychological distress. Methods: An online survey was administered from 3 January to 6 February, 2021, while a strict lockdown was in place. The sample included 288 Israelis ranging between the ages of 18–78, recruited by snowball sampling. Psychological distress was evaluated by Patients Health Questionnaire (PHQ-9) and the Generalized Anxiety Disorder questionnaire (GAD-7); attachment style by the Experiences in Close Relationships (ECR-36), and social support by the Multi-dimensional Perceived Social Support (MSPSS). A mediation model was applied with social support mediating the association between attachment style and depression and anxiety. Results: Significant correlations were found between attachment style and psychological distress, and between social support and psychological distress. Social support partially mediated the associations between attachment style and psychological distress (Depression: *p* < 0.001, confidence interval [CI] = 0.4018, 1.7468; Anxiety: *p* < 0.001, confidence interval [CI] = 0.0493, 0.9822). These results remained the same while controlling for age. Conclusion: Our findings suggest that the secure attachment style serves as a protective factor against psychological distress and vice versa; insecure attachment style serves as a risk factor for developing psychological distress during a peak period of COVID-19. Nevertheless, social support played a central role in the association between attachment style and psychological distress, thus, individuals with an insecure attachment may thus be helped by offering them social support during a crisis, which in turn may increase their well-being.

## 1. Introduction

In March 2020, WHO defined COVID-19 as a worldwide pandemic [1]. The COVID-19 outbreak has caused fatalities, economic crises, a sudden surge in unemployment, and has forced countries to adapt to new behaviors such as social distancing, which can lead to high rates of loneliness [2,3,4].

Previous data has shown that during natural disasters and pandemics such as SARS, people’s mental health is badly affected. Events of this type increase depression, anxiety, and other behavioral and psychological manifestations [5,6]. Similarly, during the COVID-19 pandemic, several studies have reported elevated levels of stress, anxiety and depression among individuals worldwide [7,8,9,10]. This has increased the risk of comorbidity during COVID-19 such as suicide and suicidal behavior [11,12,13,14,15,16]. Studies of previous pandemics such as Ebola and SARS have shown that after the outbreak is under control, there is a substantial increase in the need for psychological support [17,18].

Other studies have pointed to factors that shield individuals from psychological distress and are associated with lower levels of depression and anxiety [19,20,21]. One of the main protective factors to emerge from this literature is social support [22]. Social support is defined as the emotional, instrumental, tangible, and informational resources that individuals derive from their network ties. It is also the perception or experience that one is loved and cared for, esteemed and valued by others, and is part of a social network of mutual assistance and obligations [23,24].

Social support is crucial to individuals’ wellbeing. Deficits in social support have been widely documented to be related to psychological distress [25,26,27], whereas high social support is associated with low levels of psychological distress [28,29,30]. For example, without enough social support from family and friends, students are vulnerable to depression, stress and anxiety [25,27].

One possible explanation for these results is that social support helps people cope with stressful circumstances by increasing their engagement in beneficial coping strategies that are implemented when individuals believe their social network includes someone who is willing to listen [31,32]. Social support is considered to facilitate the development of positive self-conceptions and social skills, responsibility, competence, impulse control and prevention of social segregation which can lead to low levels of psychological distress [32].

Another main intercorrelated factor that was found to serve as a protective factor from psychological distress is attachment style. Attachment is characterized as “the propensity of human beings to make strong affectional bonds to particular others” [33]. According to Bowlby’s theory, children internalize experiences with their primary caretakers over time in such a way that early attachment relations come to form a prototype for later relationships outside the family. The quality of early attachment relationships is considered to be rooted in the extent to which the infant comes to rely on the attachment figure as a source of security [34]. Ainsworth developed a well-known laboratory procedure called “the Strange Situation” to classify infant–parent relationships (based largely on the infant’s behavior) into secure, avoidant, or anxious–ambivalent categories [34]. 

Studies suggest that children with a secure attachment form a secure working model that is characterized by a basic sense of trust that others will be dependable and available to them, especially during times of stress. By contrast, insecure working models may be described as anxious or avoidant [35,36]. Children with an anxious working model tend to have a strong desire for intimacy combined with the fear of abandonment, whereas children with an avoidant working model tend to feel discomfort with closeness due to their expectation from others to be neglectful or intrusive [35,37]. Bowlby acknowledged the importance of studying attachment processes across the lifespan and suggested that the basic functions of the attachment system continue to operate in adulthood and old age [38].

Studies have found a strong connection between attachment styles and psychological distress (i.e., depression and anxiety) during childhood and adulthood [39,40]. The results indicate that secure attachment is associated with better mental health, while insecure attachment styles are associated with higher depression and anxiety [39,40]. Secure attachment is considered to enhance the individual’s coping skills and feelings of personal worth and self-efficacy, thus reducing anxiety and fostering the development of positive, constructive strategies for dealing with environmental stressors, resulting in improved emotional adjustment [41].

In addition, attachment style is associated with social support. Several studies have indicated that individuals with insecure attachment tend to report low levels of social support, while individuals with secure attachment report high levels [42,43,44,45,46,47]. This may be due to the fact that more avoidant and more anxious individuals may lack the necessary interpersonal skills to develop strong and satisfying networks [48,49]. Individuals with insecure attachment are less likely to seek out or offer support and thus end up with a weak social support network [50,51,52]. Bowlby [53] reasoned that early attachment relationships with caregivers contribute to an individuals’ expectations of available social support and their capability to use this support when needed [52].

Thus, overall, previous studies have found that low social support is associated with high levels of psychological distress [28,29,30]. This association emerged as particularly strong during the worldwide COVID-19 outbreak [22,54,55]. Studies that have examined the relationship between attachment style and psychological distress have found that insecure attachment is associated with higher levels of depression and anxiety [39,40]. However, only a few studies have examined this association during COVID-19 [56]. Furthermore, there is little evidence as to the mediating role of social support in the association between attachment and psychological distress [57] and this potential mediation has rarely been examined during COVID-19.

The goal of the present study was to examine the relationship between social support, attachment style and psychological distress during COVID-19 in Israel and the potential mediating role of social support on the well-established relationship between attachment style and psychological distress.

The following hypotheses were made:

1. There will be a negative correlation between social support and psychological distress (i.e., depression and anxiety) during COVID-19, such that individuals with low social support will have higher levels of depression and anxiety.
2. There will be a positive correlation between attachment style and psychological distress during COVID-19, such that insecurely attached individuals will have higher levels of depression and anxiety.
3. The correlation between attachment style and psychological distress will be partially mediated by social support.

## 2. Method

Responses to questionnaires were obtained over a period of 35 days between 3 January and 6 February 2021, while the State of Israel was experiencing its third wave of COVID-19. For 42 days there were strict lockdown regulations in Israel. These included remaining within 1000 m of one’s home, prohibitions against visiting others in their homes and the banning of gatherings of more than 20 people. By this time, the number of fatalities from the pandemic in Israel had reached 3346, and the third wave had peaked with 1444 deaths in the previous month.

Digital questionnaires were administered on a Qualtrics^XM^ (https://www.qualtrics.com/ last accessed date: 6 February 2021) platform. To preserve anonymity and confidentiality, participants were not required to provide any personal or identifying information about themselves such as their name, email address or ID number. The questionnaire was also blocked at the end of its run.

### 2.1. Participants

The sample was initially composed of 395 Israeli adults, of whom 106 failed to complete two or more of the questionnaires. Of the remaining 289 participants, 60 were male and 229 were female, ranging in age from 18 to 78 (M = 37.17, SD = 14.684). One participant aged 17 was excluded from the study, leaving 59 males. The participants were recruited via advertisements posted on social networks.

### 2.2. Measures

All questionnaires were presented in randomized order to avoid systematic effects of fatigability, etc.

#### 2.2.1. Demographic Questionnaire

Personal and demographic information including age, gender and years of education was collected by self-report.

#### 2.2.2. Attachment Questionnaire

Adult attachment was measured by the Experiences in Close Relationships (ECR) developed by Brennan, Clark and Shaver [35]. The ECR is a 36-item self-report measure that assesses anxiety and avoidance, the two dimensions of attachment security. Respondents use a 7-point, partly anchored, Likert-type scale ranging from 1 (disagree strongly) to 7 (agree strongly) to respond to the items. Point 4 on the scale is anchored by neutral/mixed. Of the 36 items, 9 are reverse-scored (8 items from the Avoidance subscale and 1 item from the Anxiety subscale). Higher scores on the attachment–avoidance subscale reflect greater avoidance, higher scores on the attachment–anxiety subscale reflect greater anxiety, and low scores on these two dimensions reflect secure attachment. The validity and reliability as well as the internal consistency of the questionnaire were tested by Brennan et al. [35] and found to be high. The questionnaire was translated and adapted to Hebrew by Mikulincer and Florian [58], who found high Cronbach’s alphas in an Israeli sample (anxiety items α = 0.92; avoidance items α = 0.93). The current study found the ECR-36 to be highly reliable (Cronbach’s α: = 0.927; means and SDs are presented in Table 1).

#### 2.2.3. Depression Questionnaire

This questionnaire is composed of 9 items [59] taken from the full PHQ version [60]. The PHQ-9 questionnaire is a self-report depression screening instrument that provides both diagnostic criteria and a scale for rating the severity of depression symptoms. It contains nine questions based on the nine symptoms on the DSM–IV Criterion A for Major Depressive Episode (MDE). This questionnaire also facilitates the management of depression through the tracking of symptoms to assess the effectiveness of interventions. Items are rated on a 4-point Likert-type response format ranging from 0 not at all to 3 nearly every day on questions that pertain to mental/emotional health within the previous 2 weeks. The sum-total score for the nine questions can range from 0 to 27, where higher scores indicate greater levels or more serious levels of depression. The PHQ-9 score classifies depression severity into 4 categories: a score between 0–4 indicates a person with no or minimal depression, a score of 5–9 indicates mild depression symptoms, scores between 10–14 indicate moderate depression, 15–19 indicate moderately severe depression, and scores of 20 or higher are indicative of severe depression [59]. Reliability and validity examinations of the PHQ-9 have yielded results indicating excellent psychometric properties. The internal consistency of the PHQ-9 has been shown to be high. Similar to the high Cronbach alphas found in the PHQ primary care study (α = 0.89), the PHQ-9 also produced a high Cronbach’s alpha (α = 0.86), [59]. The PHQ-9 questionnaire was translated into Hebrew by Geulayov, Jungerman, Moses, Friedman, Miron and Gross in 2009 [61], and in their study the Cronbach’s alpha was 0.82. The current study found the PHQ-9 to be highly reliable (Cronbach’s α: = 0.879; means and SDs are presented in Table 1).

#### 2.2.4. Anxiety Questionnaire

The Generalized Anxiety Disorder questionnaire (GAD) is a self-report questionnaire that assesses symptoms of anxiety based on the DSM-IV [62]. The questionnaire administered here was the Hebrew version of the GAD-7 scale by Spitzer, Kroenke, Williams and Lowe [63]. The questionnaire contains 7 items, in which the participants are asked to state the extent to which a sentence describes them in the previous 2 weeks. Scores for all 7 items range from 0 (not at all) and 3 (nearly every day). Therefore, the total score ranges from 0–21. The total score can be categorized into four severity groups: a score between 0–4 indicates a person with no or minimal anxiety, a score of 5–9 indicates mild anxiety, scores between 10–14 indicate moderate anxiety and 15–21 indicate severe anxiety. The reliability of the questionnaire was found to be high in a validation study (α = 0.89), [62]. The current study found the GAD-7 to be highly reliable (Cronbach’s α: = 0.916; means and SDs are presented in Table 1).

#### 2.2.5. Social Support Questionnaire

The Multidimensional Perceived Social Support scale is a self-report questionnaire that examines individuals’ subjective perception of the extent of their social support. The MSPSS questionnaire is composed of 12 items developed by Ziment, Dahlem, Ziment and Farley in 1988 [64] and translated into Hebrew by Statman in 1995 [65]. The participants are asked to rate the extent to which they relate to each item on a scale from very strongly disagree (1) to very strongly agree (7). The total score can be categorized into three groups: a score between 1–2.9 indicates low social support, a score between 3–5 indicates moderate social support and a score between 5.1–7 indicates high social support. The reliability of the questionnaire as reported by the developers was high (α = 0.88), [64]. The current study found the MSPSS to be highly reliable (Cronbach’s α: = 0.938; Means and SDs are presented in Table 1).

### 2.3. Procedure

Ethical approval for this study was obtained from the Institutional Ethics Committee of The Academic College of Tel Aviv-Yafo (Approval #2020228) and all participants signed an electronic informed consent form which only then allowed access to the full set of questionnaires. Once participants signed the consent form, they were asked to complete the questionnaires online. All participants first completed a demographic questionnaire, and then the rest of the questionnaires, which appeared in a randomized order across participants.

### 2.4. Data Analysis

SPSS 27.0 for Windows was used for the statistical analysis. Categorical data were expressed as numbers and percentages, and quantitative data as the mean ± standard deviation (SD) and range. To test whether age was associated with the dependent variables (depression and anxiety), we used Pearson correlations. We transformed the predictor variable into the two categories of secure and insecure, in line with previous studies.

In addition, due to the female majority of the respondents, and the wide age range of the participants, we also examined the correlations between the study variables focusing on each sex group separately (Table 2) and the main age groups in the study (Table 3). In order to test the mediation model, we assessed confidence intervals by implementing the Hayes Process Macro for IBM SPSS Statistics 27.0, New York, NY, USA.

A retrospective power analysis to estimate the required sample size (using GPower 3.1; [66]) with an α = 0.05 and power = 0.95 indicated that the projected sample size required to detect a medium effect size (f = 0.15) was approximately N = 107. Thus, a sample size of 288 participants was satisfactory.

## 3. Results

### 3.1. Psychological Distress (i.e., Depression and Anxiety) Levels

Overall, the mean GAD-7 anxiety score was 4.83± 4.86 (ranging from 0 to 21). It broke down into 165 (57.3%) participants with minimal anxiety, 77 (26.7%) participants with mild anxiety, 28 (9.7%) participants classified as moderately anxious and 18 (6.3%) participants with severely anxious responses. For the PHQ-9 depression questionnaire, the mean score was 6.01 ± 5.35 (ranging from 0 to 27). It broke down into 31 (10.8%) participants with no depression, 110 (38.2%) participants with minimal depression, 87 (30.2%) participants with mild depression, and 34 (11.8%) participants with moderate depression, 18 (6.3%) participants with moderately high depression, and 8 (2.8%) participants with severe depression. There was a significant correlation between the GAD-7 and the PHQ-9 (*r* = 0.762 (*p* < 0.001).

### 3.2. Social Support and Attachment

Overall, the mean MSPSS social support score was 6.008 ± 1.005, ranging from 1 to 7. It broke down into 7 (2.4%) participants with low support, 33 (11.5%) participants with moderate support and 248 (86.1%) participants with high support. For the ECR-36 attachment questionnaire, the mean score was 115.73 ± 33.39 (ranging from 47 to 205). Based on the median (Md = 112), it broke down into 147 (51%) participants with secure attachment and 141 (49%) participants with insecure attachment.

### 3.3. Pearson’s Correlations among the Variables Used in the Mediation Model

To examine the relationship between the MSPSS social support score, the ECR-36 attachment style score and the GAD-7 anxiety score, Pearson correlation tests were used. For social support, there was a negative correlation with anxiety (*r*(288) = −0.258, *p* < 0.001), indicating that participants with less social support tended to be more anxious. For attachment, there was a positive correlation with anxiety (*r*(288) = 0.380, *p* < 0.001), indicating that participants with secure attachment tended to be less anxious. 

To examine the relationship between social support, attachment and the PHQ-9 depression score, a Pearson correlation test was used. For social support, there was a negative correlation with depression (*r*(288) = −0.364, *p* < 0.001), indicating that participants with less social support tended to be more depressed. For attachment there was a positive correlation with depression (*r*(288) = 0.357, *p* < 0.001), indicating that participants with secure attachment tended to be less depressed. For social support and attachment, there was a negative correlation between social support and attachment (*r*(288) = −0.311, *p* < 0.001), indicating that participants with secure attachment tended to have more social support. The bivariate correlation matrix for the variables used in the mediation model (i.e., social support, attachment, depression, and anxiety) is presented in Table 4 and supported the predicted relationships across variables.

### 3.4. Mediation Model

**Figure 1:**
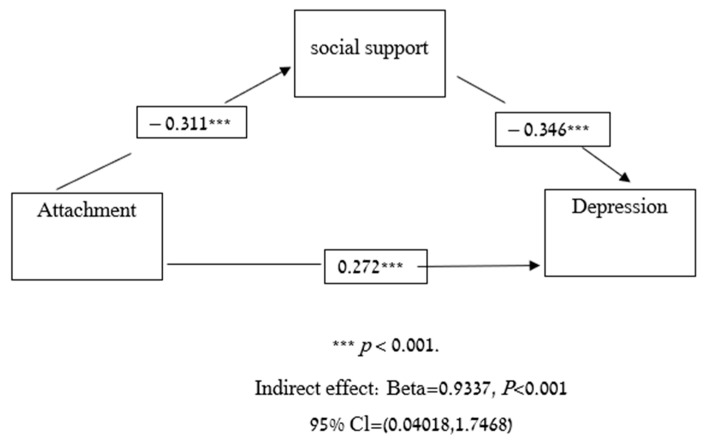
Mediation model-attachment, social support and depression.

**Figure 2:**
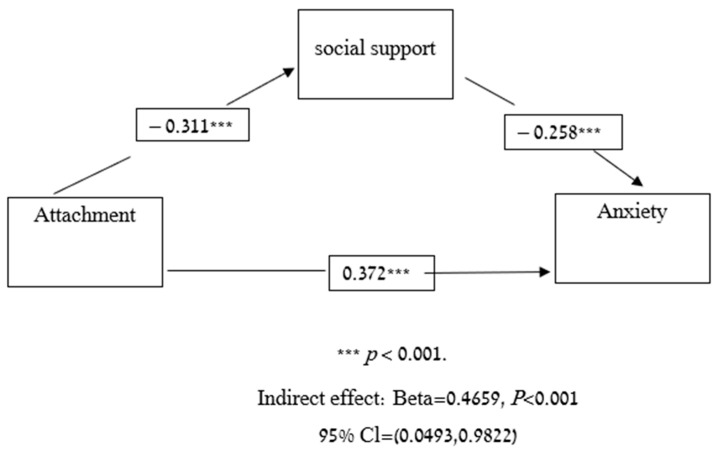
Mediation model-attachment, social support and anxiety.

Next, we used a mediation analysis to test whether social support could partially explain the associations between attachment style and depression. As hypothesized, social support partially mediated the association between attachment and depression (*p* < 0.001, confidence interval [CI] = 0.4018, 1.7468) (Figure 1). In order to test whether social support could partially explain the associations between attachment and anxiety we also used a mediation analysis. As hypothesized, social support partially mediated the association between attachment and anxiety (*p* < 0.001, confidence interval [CI] = 0.0493, 0.9822) (Figure 2).

### 3.5. Further Analyses

To determine whether age was associated with the dependent variables (i.e., anxiety and depression) we used Pearson correlations. The Pearson correlation between age and PHQ-9 was significant (*r*= −0.231 (*p* < 0.001). Similarly, a Pearson correlation between age and GAD-7 was significant as well (*r*= −0.221 (*p* < 0.001). To better understand this association, a further correlation analysis was conducted while controlling for age, which revealed a partial correlation: *r* = −0.413, *p* < 0.001. When testing the correlation between social support and anxiety while controlling for age, there was a partial correlation: *r* = −0.278, *p* < 0.001. In addition, when testing the correlation between attachment and depression while controlling for age, there was a partial correlation: *r* = 0.335, *p* < 0.001. When testing the correlation between attachment and anxiety while controlling for age, there was a partial correlation: *r* = 0.362, *p* < 0.001. The correlation between attachment and depression while controlling for social support and age remained significant in a way that corresponded to the mediation analysis: *r *= 0.236, *p* < 0.001. Finally, the correlation between attachment and anxiety while controlling for social support and age was also significant in a way that corresponded to the mediation analysis: *r* = 0.297, *p* < 0.001. These findings suggest that even though there were significant associations between age and psychological distress, when we controlled for age, these associations remained significant.

## 4. Discussion

To better understand the associations between attachment style, psychological distress (i.e., depression and anxiety) and social support during COVID-19 pandemic, the relationships between these variables as well as a mediation model were examined, with social support as a mediator. Specifically, we examine whether insecure attachment style and low social support would be related to high levels of depression and anxiety during the COVID-19 pandemic. Moreover, we examined whether social support mediated the well-documented relationships between attachment style and psychological distress.

As hypothesized, low social support was correlated with more symptoms of depression and anxiety, and similar findings were also found among participants classified as having an insecure attachment style. These findings are consistent with studies that reported a strong association between attachment styles and psychological distress as well as between social support and psychological distress [28,29,30,39,40]. One explanation for these findings may be that both high social support and secure attachment style (each in its own way, in that social support implies the belief that someone is willing to listen, and secure attachment style enhances one’s personal worth and self-efficacy) help to cope with stressful circumstances by increasing the use of beneficial engagement coping strategies. Hence, especially during stressful and unusual times such as the COVID-19 pandemic, social services should concentrate on individuals with low social support/insecure attachment style since both serve as a risk factors for suffering from psychological distress.

In addition, as hypothesized, during COVID-19 the correlations between attachment style and psychological distress were partially mediated by social support. These findings are also consistent with studies conducted during noncrisis periods [57]. These findings may show that individuals with insecure attachment style may lack the necessary interpersonal skills to develop strong and satisfying social networks [48,49]. In addition, these individuals may have lower expectations for available social support and less capability to use this support when needed [53]. Attachment style is developed and established in infancy and continues to affect individuals’ well-being throughout their life span [38,39,40]. In the current study, the association between attachment style and psychological distress was partially mediated by social support. These findings suggest that, even with an insecure attachment style, the presence of social support may “protect” those individuals and thus improve their well-being. This result can be harnessed to help insecurely attached individuals by enabling them to develop social skills/ providing them with social support in times of crisis, which in turn can, at least partially, help them experience less depression and anxiety.

There are number of limitations to this study. First, it was conducted in Israel during the third wave of COVID-19 pandemic at the start of the vaccination campaign. The presence of a possible solution may have affected the mental health of individuals in Israel at this period of time, which may have affected the results. Second, in this study we used the MSPSS questionnaire which evaluates social support and not attachment style but overlaps with the ECR-36 on numerous questions. This overlap may have led to biased results in the mediation model. It is possible that using a social support questionnaire with less overlap with the ECR-36 questionnaire could lead to a better evaluation of this variable. Additionally, the study included relatively small number of respondents, while using a bigger number of respondents could contribute to a more representative sample. Finally, since our study mainly examined whether those mediated relations exist, the mechanisms underline these relations still remain unclear.

## 5. Conclusions

Our findings suggest that secure attachment style serves as a protective factor against psychological distress and, vice versa, insecure attachment style serves as a risk factor for developing psychological distress during a peak period of COVID-19. Nevertheless, social support played a central role in the association between attachment style and psychological distress; thus, individuals with an insecure attachment may be helped by offering them social support during crises, which in turn may increase their well-being.

The findings demonstrate that during the COVID-19 period, levels of depression and anxiety among the Israeli population were high, especially among individuals with insecure attachment style and those with poorer social support (compared to individuals with secure attachment style and individuals with greater social support). In addition, although there was a correlation between attachment style and psychological distress, this relationship was partially mediated by social support. This interconnection may be related to the fact that individuals with insecure attachment style have not developed close relationships that enable social support, and they feel unable to seek help when needed. The findings thus point to the importance of the role of social support and attachment style for mental well-being in complex times such as the COVID-19 period. This study is one of few in general, and during the COVID-19 period in particular, that sheds light on the mediating role of social support in the relationship between attachment style and psychological distress.

As noted, since epidemics and crises are known to have long term psychological effects that persist after the end of the crisis [17,18], it is important to examine the relationships reported in the present study with depression and anxiety even after the COVID-19 period, to assess their long-term stability. In addition, since there is almost no research on the mediated relationship found in this study that can have practical use (by assisting individuals with insecure attachment style to improve their well-being), we encourage future studies to further investigate this mediation to increase its external validity.

## Figures and Tables

**Table 1:** Descriptive statistics of psychological measures.

Questionnaire	M	SD
ECR-36 (36–252)	115.725	33.392
PHQ-9 (0–27)	6.017	5.356
GAD-7 (0–21)	4.833	4.867
MSPSS (1–7)	6.008	1.005

Note: ECR-36 = Experiences in Close Relationships; PHQ-9 = Patient Health Questionnaire; GAD-7 = Generalized Anxiety Disorder questionnaire; MSPSS = Multidimensional Perceived Social Support scale.

**Table 2:** Pearson correlations between the variables used in the mediation model dividing by sex.

SEX	Questionnaire	MSPSS	ECR-36	PHQ-9	GAD-7
Male	MSPSS	--	--	--	--
	ECR-36	−0.311 ***	--	--	--
	PHQ-9	−0.224	0.236	--	--
	GAD-7	−0.158	0.273 *	--	--
Female	MSPSS	--	--	--	--
	ECR-36	−0.311 ***	--	--	--
	PHQ-9	−0.402 **	0.390 **	--	--
	GAD-7	−0.288 **	0.418 **	--	--

Note: ECR-36 = Experiences in Close Relationships; PHQ-9 = Patient Health Questionnaire; GAD-7 = Generalized Anxiety Disorder questionnaire; MSPSS = Multidimensional Perceived Social Support scale * *p* < 0.05 ** *p* < 0.01. *** *p* < 0.001.

**Table 3:** Pearson correlations between the variables used in the mediation model dividing by main age groups.

Age	Questionnaire	MSPSS	ECR-36	PHQ-9	GAD-7
18–27	MSPSS	--	--	--	--
	ECR-36	−0.311 ***	--	--	--
	PHQ-9	−0.478 **	0.381 **	--	--
	GAD-7	−0.357 **	0.345 **	--	--
38–47	MSPSS	--	--	--	--
	ECR-36	−0.311 ***	--	--	--
	PHQ-9	−0.362 *	0.355 *	--	--
	GAD-7	−0.369 **	0.469 **	--	--
48–57	MSPSS	--	--	--	--
	ECR-36	−0.311 ***	--	--	--
	PHQ-9	−0.462 **	0.299 *	--	--
	GAD-7	−0.189	0.400 **	--	--

Note: ECR-36 = Experiences in Close Relationships; PHQ-9 = Patient Health Questionnaire; GAD-7 = Generalized Anxiety Disorder questionnaire; MSPSS = Multidimensional Perceived Social Support scale * *p* < 0.05 ** *p* < 0.01. *** *p* < 0.001.

**Table 4:** Pearson correlations between the variables used in the mediation model.

Questionnaire	MSPSS	ECR-36	PHQ-9	GAD-7
MSPSS	--	--	--	--
ECR-36	−0.311 ***	--	--	--
PHQ-9	−0.364 ***	0.357 ***	--	--
GAD-7	−0.258 ***	0.380 ***	--	--

Note: ECR-36 = Experiences in Close Relationships; PHQ-9 = Patient Health Questionnaire; GAD-7 = Generalized Anxiety Disorder questionnaire; MSPSS = Multidimensional Perceived Social Support scale *** *p* < 0.001.

## Data Availability

The data presented in this study are available on request from the corresponding author. The data are not publicly available due to restrictions as privacy.
